# Influence of Backfat Thickness and the Interval from Altrenogest Withdrawal to Estrus on Reproductive Performance of Gilts

**DOI:** 10.3390/ani11051348

**Published:** 2021-05-10

**Authors:** Krittawat Thitachot, Voramet Sirinopwong, Viriya Seemuang, Akkapon Ratchatasriprasert, Roy N. Kirkwood, Nutthee Am-in

**Affiliations:** 1 Faculty of Veterinary Science, Chulalongkorn University, Bangkok 10330, Thailand; krittawat.t@student.chula.ac.th (K.T.); voramet.si@student.chula.ac.th (V.S.); viriya.s@student.chula.ac.th (V.S.); akkapon.r@student.chula.ac.th (A.R.); 2 School of Animal and Veterinary Sciences, University of Adelaide, Roseworthy, SA 5371, Australia; roy.kirkwood@adelaide.edu.au; 3 Department of Obstetrics, Gynaecology and Reproduction, Faculty of Veterinary Science, Chulalongkorn University, Bangkok 10330, Thailand; 4 Swine Reproduction Research Unit, Chulalongkorn University, Bangkok 10330, Thailand

**Keywords:** gilts, altrenogest, backfat depth, fertility

## Abstract

**Simple Summary:**

Altrenogest, also known as allyl trenbolone, is a steroidal progestin that is widely used in veterinary medicine to synchronize estrus in gilts. To achieve the target number of services per week, enough weaned sows and gilts are needed each week for breeding. A problem with progestogen-synchronized replacement gilts is the variation of the interval between last feeding of altrenogest and onset of estrus. In the present study, we found that gilt backfat thickness had a strong positive correlation with the interval between last feeding of altrenogest and onset of estrus. This result may come from a larger reservoir of altrenogest in adipose tissue in fatter gilts.

**Abstract:**

Estrus synchronization of gilts can be achieved by feeding the orally active progestogen altrenogest (AT) to cycling gilts at 20 mg/day for 14 to 18 days with gilts usually returning to estrus 4 to 8 days after the last feeding. In practice, gilts failing to exhibit estrus by 6 days after AT withdrawal may compromise weekly breeding targets. The cause of prolonged intervals to estrus are unknown but may involve prolonged suppression due to the release of progesterone (P4), and by extension AT, from adipose tissues. The present study examined relationships between gilt P2 backfat depth (<13.5 mm, 14–16.5 mm, >17 mm groups), the AT withdrawal to estrus interval, and subsequent reproductive performance in gilts. We noted longer intervals to estrus in gilts with greater P2 backfat depths (*p* < 0.0001), and higher serum P4 concentrations on the last day of AT feeding and at estrus detection (*p* < 0.05). Total born litter sizes were unaffected by backfat depth, but pigs born alive progressively decreased with increasing backfat depth with the fattest gilts producing the fewest liveborn pigs (*p* < 0.05). Taken together, these data suggest that adipose tissues may provide a reservoir of steroid, with its release from fatter gilts having potential negative effects on their subsequent reproductive performance.

## 1. Introduction

Predictability of weaner output is largely controlled by breeding enough females each week and, where insufficient weaned or return sows are available, the gilt pool must make up the shortfall [1]. To improve timely availability of service-ready gilts, estrus synchronization of groups of randomly cycling gilts is practiced by the feeding of the orally active progestogen altrenogest, typically at 15 to 20 mg/day for 14 to 18 days [2,3]. Synchronising gilts with altrenogest is associated with an increased ovulation rate [4] and subsequent litter size [5] from breeding at the subsequent estrus.

The altrenogest provides an artificial luteal phase via suppression of gonadotro-phins, particularly LH, and following the withdrawal of altrenogest from primiparous sows the LH pulsatility recovers after several hours, the timing depending on the duration of feeding. With recovery of LH pulsatility the follicular phase is initiated, and most gilts will exhibit estrus 4 to 6 days later [6]. This variability in the interval to the start of follicular phase, and the innate variability in duration of the follicular phase, results in variation of the interval between last feeding of altrenogest and onset of estrus. Indeed, following feeding of altrenogest at 20 mg/day for 18 days, 77% of gilts returned to estrus in 4–6 days and 23% in 7–8 days (Nutthee Am-in, unpublished data). The approximately 23% of gilts returning to estrus in 7–8 days were not service-ready during their designated breeding week.

A relationship between progesterone concentrations in blood and backfat tissue during the estrous cycle has been reported [7]. These authors documented an “enormous storage” of progesterone in backfat (approximately 0.9 ng/mg fat) that showed a 2 day delay in reduction in concentrations following luteolysis compared to plasma concentrations, with backfat concentrations remaining considerably higher than in plasma throughout the cycle, including the estrous period. Therefore, to test the hypothesis that backfat depth will affect return to estrus in altrenogest-synchronized gilts, we examined the relationship between backfat thickness, and presumably steroid stores available for release, and the interval from altrenogest withdrawal to estrus and subsequent reproductive performance of cyclic gilts synchronized using altrenogest.

## 2. Materials and Methods

A total of 90 Landrace x Yorkshire gilts were exposed to a boar from 23 weeks of age to detect their pubertal estrus. The pubertal gilts were accommodated in an open housing system allowing 1.8 m^2^ per gilt. Commercially formulated feed was provided, allowing 2.0 kg/gilt/day with water provided ad libitum via nipple drinkers in the pen. When the youngest pubertal gilt achieved 210 days of age, all gilts were weighed and then individually orally dosed with 20 mg/day altrenogest (AT; Altresyn^®^; CEVA, Libourne, France) for 18 days consecutively, regardless of stage of their cycle. At the time of first AT dose, age, weight, and average daily gain (ADG) from weaning were recorded, and P2 backfat depths (65 mm off midline at the level of the last rib) measured using A-mode ultrasound (Renco lean meater^®^, Minneapolis, MN, USA).

From the day following altrenogest withdrawal, all gilts received fenceline contact with mature boars for 15 min twice daily and the last altrenogest dose to estrus interval (hereafter referred to as interval to estrus) recorded. At detection of estrus and 24 h later gilts were artificially inseminated with 3 × 10^9^ sperm in 80 mL extender. Gilts went to term and farrowing rates and litter sizes were recorded.

On days 0 and 18 of altrenogest treatment, and at detection of the post-treatment estrus, each gilt was blood sampled by jugular venipuncture and serum harvested and stored at −20 °C until required for assay of progesterone. Serum progesterone concentrations were determined by ELISA as described by Tummaruk et al. [8]. Assay sensitivity and intra- and inter-assay coefficients of variance were 0.06 ng/mL, 1.64%, and 2.08%, respectively.

All statistical analyses were performed using SAS 9.4 (SAS Institute Inc., Cary, NC, USA). Gilt age at start of the study, their ADG from weaning to first altrenogest dose, backfat depth at first altrenogest dose, and serum progesterone concentrations, were analyzed for relationships to interval to estrus and subsequent litter size using Pearson’s correlation.

The gilts were retrospectively assigned to 3 groups by back fat depths of <13.5 mm, 14–16.5 mm, and >17 mm [9] or AT to estrus intervals of ≤5 days, 6 or 7 days, and ≥8 days, and associations with pubertal age, interval to estrus, and litter size examined by ANOVA, and farrowing rates were compared by chi-square. Differences were deemed significant at *p* ≤ 0.05.

## 3. Results

There were no significant effects of age, ADG, or serum progesterone concentrations at first altrenogest treatment on the interval to estrus (Table 1). However, backfat depth at first altrenogest dose (*p* < 0.0001), and progesterone concentrations at final altrenogest dosing, and at estrus detection were positively associated with the interval to estrus (*p* < 0.05; Table 1). Age at puberty was older (*p* < 0.05) and the interval to estrus was longer (*p* < 0.05) for fatter gilts (>17 mm) than for their leaner contemporaries (Table 2).

In this study, total and live born litter sizes were not significantly correlated with gilt age at first altrenogest dose or their ADG, nor with their progesterone concentrations on days 0, 18 or at estrus detection (Table 1). Similarly, total born litter size was not influenced by backfat depth at first AT dose (Table 2). However, there was a negative correlation between the backfat depth and number of piglets born alive (*p* < 0.03, Table 1), there being a progressive decrease in live born litter size with increasing backfat depth (Table 2).

The farrowing rate of gilts with 14 to 16.5 mm backfat depth was higher than for the leaner and fatter gilts (*p* < 0.05; Table 2). Further, farrowing rate and both total and live born litter sizes were reduced in gilts with the longest interval to estrus (Table 3).

## 4. Discussion

Our results demonstrated a lack of effect of breeding age, and weight as indicated by their ADG, on gilt fertility. This is consistent with previous data demonstrating that, compared to pubertal breeding, gilt fertility improved when bred at second estrus, but little further improvement is accrued by breeding at a third or subsequent estrus [10]. Our gilts were bred at their second or later estrus, so an effect of age or weight per se on gilt reproductive performance would not be anticipated.

The results of the present study do clearly indicate an influence of gilt backfat depth, and thus presumably total progesterone and AT storage capacity, on their reproductive performance. The positive association between serum progesterone concentrations and the interval to estrus is interesting. Potentially, this may reflect the unknown distribution of day of the estrous cycle when altrenogest feeding commenced with some gilts undergoing luteolysis very soon before, or after, the last altrenogest treatment, resulting greater progesterone and altrenogest residual storage and so a longer period of LH suppression prior to initiation of the follicular phase. While this suggestion cannot be discounted, there is considerable literature indicating that 18 days of altrenogest treatment should allow all gilts to achieve luteolysis prior to the final altrenogest treatment [4,11,12]. However, to our knowledge, current research on adipose tissue steroid storage and subsequent release profiles after luteolysis is not available. There was also a negative association between backfat depth and live born, but not total born, litter size. The etiology of this association is unclear, although an effect on stillbirth rate is evident. However, given that backfat depth was positively correlated with the interval to estrus, and that interval to estrus was negatively associated with litter size variables, the impact of backfat depth on litter size may have been indirect. Alternatively, if these fatter gilts were also fatter at farrowing, this could impact stillbirth rates via higher progesterone concentrations increasing farrowing durations. However, in the absence of data on backfat depth at farrowing and durations of farrowing, this suggestion remains speculative. Even if this is accepted, backfat depth and possible increased circulating progesterone concentrations are not the only determinants of sow farrowing performance. Other factors, including housing management such as crates vs. pens vs. free farrowing, may also impact farrowing duration and associated stillbirth rate.

It is interesting that both the fattest and leanest gilts had significantly lower farrowing rates than those gilts having 14.0 to 16.5 mm P2 backfat depth. The greater backfat depths and increased interval to estrus interval may result in more aged, and so poorer quality, oocytes. If this suggestion is accepted, then the resultant poorer subsequent embryo quality would be expected to reduce farrowing rate. Additionally, it is noted that the fatter gilts were also the oldest at puberty and, as such, may be innately less fertile [10]. Although a reduced total born litter size could also be anticipated in these fatter gilts, this was not evident. However, lower farrowing rates and smaller litters are not inextricably linked. The etiology of a lower farrowing rate in the leanest gilts is not immediately evident. However, although speculative, it is possible that the leaner gilts were physiologically less mature, and their relative immaturity was expressed in poorer fertility. In this regard, we cannot discount that a mixture of earlier and later maturing gilts were involved in our study, and that less mature gilts may be both leaner and innately less fertile [13].

An effect of interval to estrus on gilt fertility was clearly evident. Interestingly, gilts with the longest intervals to estrus were the oldest at puberty, had the lowest farrowing rate, and the smallest total born and live born litter sizes. The underlying etiology is unclear but out results suggest a potential multifactorial effect. Fatness affects interval to estrus but not total born litter size, although the fattest gilts were the oldest at puberty. Taken together, our data clearly indicate a relationship between backfat depth and fertility of gilts estrus-synchronized using altrenogest. Our data are consistent with a role for release of progesterone or altrenogest from adipose tissue stores on gilt fertility. The etiology of this impact on fertility remains to be determined, but an effect on oocyte quality is possible. Future studies could examine the relationship between fatness, gilt fertility, and circulating concentrations of anti-Mullerian hormone (AMH), since we have recently documented that earlier maturing gilts had higher serum AMH concentrations than did later maturing gilts and also an improved ovarian response to exogenous gonadotrophins [14]. 

## 5. Conclusions

Our data support the hypothesis that the backfat depth affects return to estrus in altrenogest-synchronized gilts, we found the relationship between backfat thickness, and presumably steroid stores available for release. The gilt backfat thickness has a strong positive correlation with the interval between last feeding of altrenogest and onset of estrus. These data may be used to predict onset of estrus after last feeding of altrenogest and help pig farmers to manage the target number of gilts in each batch.

## Figures and Tables

**Table 1:** Correlation of age of gilts, average daily gain (ADG), serum progesterone (P4) concentration and backfat thickness with the altrenogest (AT) withdrawal to estrus interval and litter size.

	AT Withdrawal to Estrus		Total Born Piglets		Live Born Piglets	
	r	*p*	r	*p*	r	*p*
Age at puberty	0.5	0.06	0.3	0.09	0.4	0.06
Age at first AT feed	−0.1	0.2	0.09	0.3	0.1	0.4
ADG wean to first AT feed	−0.1	0.1	0.2	0.1	0.2	0.4
Serum P4 at first AT feed	0.08	0.4	0.1	0.2	0.1	0.09
Serum P4 at last AT feed	0.2	0.05	0.3	0.4	0.1	0.4
Serum P4 at first insemination	0.3	0.04	0.1	0.4	0.3	0.2
Backfat depth at first AT feed	0.5	0.0001	0.2	0.3	−0.2	0.03

**Table 2:** Influence of backfat depth at first AT feed on gilt performance (mean ± SD).

	Backfat Depth (n)		
	<13.5 mm (n = 29)	14–16.5 mm (n = 34)	>17.0 mm (n = 27)
Age at puberty, days	179.9 ± 11.5 ^a^	188.25 ± 15.9 ^a^	211.6 ± 10.1 ^b^
Age at first AT feed, days	218.1 ± 12.8 ^a^	213.6 ± 14.2 ^a^	215.6 ± 9.5 ^a^
AT withdrawal to estrus, days	5.4 ± 1.7 ^a^	5.2 ± 1.8 ^a^	7.0 ± 1.7 ^b^
Farrowing rate, %	70.6 ^a^	85.7 ^b^	76.3 ^a^
Total born piglets	11.5 ± 1.5 ^a^	11.6 ± 1.5 ^a^	11.6 ± 1.8 ^a^
Live born piglets	10.8 ± 1.5 ^a^	10.1 ± 1.8 ^a^	9.6 ± 2.0 ^b^

Means with different superscript within the same parameter differ, *p* < 0.05.

**Table 3:** Influence of interval from AT withdrawal to estrus on gilt performance (mean ± SD).

	Altrenogest to Estrus Interval (n)		
	≤5 days (n = 40)	6–7 days (n = 38)	≥8 days (n = 12)
Age at puberty, days	189.9 ± 11.4 ^a^	203.8 ± 10.6 ^ab^	213.8 ± 12.3 ^b^
Age at first AT feed, days	219.1 ± 13.2 ^a^	214.2 ± 11.2 ^a^	217.6 ± 8.9 ^a^
Farrowing rate, %	81.6 ^a^	81.5 ^a^	64.1 ^b^
Total born piglets	11.6 ± 1.3 ^a^	11.4 ± 1.1 ^a^	10.6 ± 1.4 ^b^
Live born piglets	10.9 ± 1.5 ^a^	10.8 ± 1.3 ^a^	9.4 ± 1.0 ^b^

Means with different superscript within the same parameter differ, *p* < 0.05.

## Data Availability

Not applicable.
